# Editorial: Perspectives on the “Bilingual Advantage”: Challenges and Opportunities

**DOI:** 10.3389/fpsyg.2019.01346

**Published:** 2019-06-06

**Authors:** Peter Bright, Roberto Filippi

**Affiliations:** ^1^Department of Psychology, Anglia Ruskin University, Cambridge, United Kingdom; ^2^Institute of Education, University College London, London, United Kingdom

**Keywords:** multilingualism, cognitive control, executive functions, bilingual advantage, bilingualism

When we ask our students or members of the general public the question *Is being bilingual/multilingual an advantage?* The answer, invariably, is yes. The reasons provided are intuitively sensible and leave little room for disagreement. Multilingual speakers can communicate with different people, they understand different cultures, they have more job opportunities, they can travel the world with more confidence, and so forth.

However, when we formulate the question in a different way, *Is being bilingual/multilingual an advantage for cognitive development?* Answers are not as straightforward. Some are concerned that second language learning may delay language acquisition in early stages of life, others think that children should focus more on one language to avoid mental confusion. In some cases, and this is probably the most disturbing situation, education professionals advise parents from different cultures to raise their children as monolinguals, advocating that this is more likely to lead to good academic achievement (e.g., Festman et al., 2017). This opinion almost certainly derives in part from early evidence (e.g., Saer, 1923) for a mental delay in bilingual children compared to monolingual peers on a range of tests measuring intelligence quotient (IQ).

The more recent work of pioneer scientists (e.g., Peal and Lambert, 1962; Bialystok and Ellen, 1991), incorporating more rigorous and systematic paradigms and procedures, has underpinned a now widely-held consensus among researchers in the field, that multilanguage learning is not detrimental for cognitive development. Nevertheless, while few—if any—scientists now hold the position that multilanguage acquisition underpins a cognitive *disadvantage*, there is ongoing vigorous debate about whether there are distinct cognitive *advantages* associated with multilingualism that cannot be explained by other candidate explanatory variables. Understanding the cognitive sequelae of bilingualism presents many hurdles that will require continued intense effort.

Collectively, the 17 articles contained herein, reflect the current state of the field, with well-defended positions on opposing sides of current debate. Altogether, 44 leading scientists in the field of multilingualism have contributed with commentaries, meta-analyses, methodological advice, and empirical research. We are most grateful to them, to the independent reviewers and to Frontiers for providing the means to make this happen.

Yu and Schwieter begin this collection with a conceptual analysis of the significance of language mode in bilingual cognition, that is, the degree of co-activation of the two languages at any one time (Grosjean, 1998, 2010). They encourage more robust and systematic consideration of language mode in future studies due to its potential modulatory effect on language activation and also, therefore, on the likely cognitive benefits associated with bilingualism. In a short review, these authors provide a convincing case that the failure to assess and control language mode may, at least in part, explain the contradictory findings reported in the literature. The controversy about whether, and the extent to which, bilingualism confers cognitive benefits is also tackled by Takahesu Tabori et al. in their timely methodological review which, in particular, addresses sample characteristics. They argue that most published studies provide insufficient information on language experience/background, social context of language use and decry the paucity of longitudinal designs which, they argue, offer a greater degree of experimental control. They encourage work toward more widely agreed criteria for terms such as “native language,” “first language,” “second language,” etc., and argue against over-simplification, most obvious in the long-standing dichotomised categorization of monolingual vs. bilingual and bilingual advantage vs. no advantage. Several of the studies in this collection demonstrate a shift to more nuanced and precise conceptualization of bilingual cognition, and this, of course, is to be welcomed and encouraged.

In her excellent review, Incera, considers timing of processing in the bilingual mind as a tool for understanding how bilingual and monolingual cognition may diverge. She offers a range of recommendations for future attempts at resolving conflicting findings, and researchers would do well to act on them. Of these, inclusion of time-sensitive measures and baseline conditions, consideration of bilingualism as a continuous variable and a focus on group by condition interactions over main effects of bilingualism are, in our view, most likely to lead to sustained theoretical advances in this area. Hernandez et al. outline a neuroemergentist approach which, they argue, may also offer a more ambitious and plausible framing of the complex ways in which bilingualism may interact with development of domain-general cognitive control.

Schroeder tackles the possibility that bilingual children have an advantage in theory of mind, presenting a meta-analysis of 16 studies. Small to medium positive effects of bilingualism were observed (contingent on the analysis), indicating that second language learning may have modest implications for the development of social competence, although well-grounded explanations for this association are currently lacking.

Five studies address the impact of multi-language experience on cognitive control in infants or children. Mercure et al. explored attention to still faces in monolingual infants, unimodal bilingual infants (i.e., learning two spoken languages) and bimodal bilingual infants of Deaf mothers (learning British Sign Language and spoken English). Equivalent attention capture and maintenance by face stimuli was observed in monolinguals and bimodal bilinguals, but unimodal bilinguals showed comparatively faster attention capture and maintenance, raising implications of multilanguage learning for social communication during infancy. Poarch provides a replication study with findings partly consistent with the central claim of the bilingual advantage theory, that controlling multiple languages in daily life confers genuine benefits in domain-general cognitive control. Specifically, equivalent performance among monolingual and bilingual children was observed on the Simon task, but the bilinguals demonstrated a significant advantage on the flanker task, indicating that these tasks may recruit partly distinct mechanisms of cognitive control that are differentially sensitive to language environment and may also follow different developmental trajectories. Struys et al. also employed the Simon and flanker tasks in a comparison of performance among younger and older monolingual and bilingual Dutch-French children. They report equivalent performance across language groups but, crucially, there was marked variation in the actual strategies employed to resolve conflict in the tasks. This finding is consistent with recent (currently unpublished) work from our lab which indicates significant differences in the neural networks recruited among bilingual and monolingual participants when resolving conflict despite the absence of any group effects at the behavioral level.

Janus and Bialystok consider the reported association between executive function and emotion regulation, arguing that bilingual advantages in executive control may, intuitively, also underpin performance benefits in emotional contexts. However, in their study of emotional face N-back task performance in monolingual and bilingual children, there were no group differences in the overall effect of emotional valence on reaction time (despite better accuracy in bilinguals). Czapka et al. present a novel and intriguing study of real word and non-word spelling in monolingual and bilingual third grade (~9 year-old) primary school children in Germany, providing compelling evidence that monolinguals at this age are better able to deploy higher level cognitive control during spelling, most likely due to superior knowledge of the German language. For bilinguals, German lexicon size was a better predictor of spelling ability than executive function. These findings reinforce the importance of adopting a fine-grained, developmentally informed approach to charting interactions between multi-language learning and cognitive development, without which we are unlikely to resolve the contradictory claims and entrenched positions so prevalent in the recent literature.

Seven studies examine bilingual processing in adults, each of which focuses on a key issue in current debate. Naeem et al. address the potential importance of an alternative explanatory variable: socioeconomic status (SES). Employing demonstrably low and high SES monolingual and bilingual participants, these authors found evidence (from Simon task performance) that bilingualism may promote a speed of processing advantage, but only in those with low SES. Furthermore, there was no evidence for a bilingual advantage in executive planning ability (based on Tower of London performance), with monolinguals showing a disproportionate advantage. Van der Linden et al. explore interference suppression, response inhibition, and short-term memory performance in professional simultaneous interpreters. To the extent that bilingual cognitive advantages are associated with the requirement to manage and control simultaneously active languages in daily life, the authors argue that a comparison of such highly skilled bilinguals against monolinguals should increase the likelihood of detecting a bilingual advantage, if it exists. In fact, the two groups performed similarly on all measures (flanker, Simon, and digit span tasks), a finding reinforced in a second experiment which incorporated an additional group of second language teachers. Despite anecdotal evidence for an STM advantage over monolinguals among interpreters, this evidence is clearly difficult to reconcile with bilingual advantage theory. In their study on the effect of language similarity on the association between linguistic performance and executive function, Oschwald et al. found very limited evidence for benefits in executive function associated with the increased demands of managing more dissimilar languages. These results, therefore, also offer evidence against the claim that managing cross-language interference promotes or enhances executive function. Evidence presented by Borragan et al. provides a possible explanation for lack of transfer from control of language interference to non-verbal executive function. These authors examine performance in highly proficient but unbalanced bilinguals on a multilingual rapid picture naming task incorporating multiple inhibitory demands. Findings are most consistent with the existence of functionally independent inhibitory mechanisms associated with language processing which may not be recruited in non-verbal tasks.

Further evidence against the existence of a genuine bilingual advantage, either in attentional control or response inhibition is presented by Paap et al. In this study, no effects attributable to bilingualism were observed on the tasks whether (i) participants were separated into monolingual or bilingual groups or (ii) degree of bilingualism was treated as a continuous variable, and Bayes factor analyses robustly supported the null hypothesis. The study by Goldsmith and Morton tests recent evidence by Grundy et al. (2017) that bilingual adults show smaller sequential congruency effects than monolingual adults, perhaps consistent with a bilingual efficiency advantage in the disengagement of attention from no longer relevant task stimuli. This new study, offered as a replication, showed statistically equivalent performance in both groups. However, Grundy and Bialystok have published a reply in *Frontiers* (available here), outlining that the study is not a direct replication but differs in several ways. Perhaps, most importantly, they point out that Goldsmith and Morton employ long rather than short response-to-stimulus intervals, and it is at short intervals that language group differences in the disengagement of attention can most readily be observed.

The possibility that bilingualism may offer protection against age-related cognitive deterioration and/or neural degeneration is an important issue in the literature. Rather than addressing vocabulary, syntax, or comprehension, Sundaray et al. take the novel approach of addressing non-literal language (pragmatic inference making) in young and older monolingual and bilingual participants. With the exception of conventional metaphors (for which an age-related deficit was observed only in monolinguals) no differences between language groups in processing pragmatic inferences were observed. Thus, the evidence here suggests a possible protective effect of bilingualism in comprehension of non-literal language, restricted to conventional metaphors.

There are many challenges in this line of research, but when there is challenge there should also be opportunity to advance knowledge. In collecting these articles within a single volume, we hope readers will take the opportunity to digest the full range of empirically supported inferences, and further develop a well-informed understanding of how (and the extent to which) the process of acquiring a second language confers domain general cognitive benefits.

## Author Contributions

All authors listed have made a substantial, direct and intellectual contribution to the work, and approved it for publication.

### Conflict of Interest Statement

The authors declare that the research was conducted in the absence of any commercial or financial relationships that could be construed as a potential conflict of interest.
